# *N*′-(4-Cyano­benzyl­idene)furan-2-carbohydrazide monohydrate

**DOI:** 10.1107/S1600536810022221

**Published:** 2010-06-16

**Authors:** Yu-Feng Li, Fang-Fang Jian

**Affiliations:** a Microscale Science Institute, Department of Chemistry and Chemical Engineering, Weifang University, Weifang 261061, People’s Republic of China

## Abstract

In the title compound, C_13_H_9_N_3_O_2_·H_2_O, the dihedral angle between the aromatic rings is 10.7 (4)° and an intra­molecular N—H⋯O hydrogen bond occurs. In the crystal, the components are linked by N—H⋯O, O—H⋯N and O—H⋯O hydrogen bonds.

## Related literature

For a related structure and background references, see: Li *et al.* (2010 ▶).
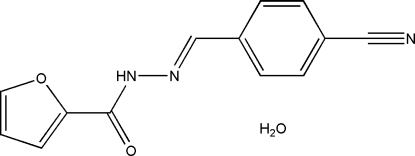


## Experimental

### 

#### Crystal data

C_13_H_9_N_3_O_2_·H_2_O
*M* *_r_* = 257.25
Monoclinic, 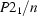

*a* = 7.0501 (14) Å
*b* = 14.295 (3) Å
*c* = 12.640 (3) Å
β = 103.38 (3)°
*V* = 1239.3 (4) Å^3^
*Z* = 4
Mo *K*α radiation
μ = 0.10 mm^−1^
*T* = 293 K
0.22 × 0.20 × 0.18 mm

#### Data collection

Bruker SMART CCD diffractometer
11389 measured reflections
2834 independent reflections
1568 reflections with *I* > 2σ(*I*)
*R*_int_ = 0.043

#### Refinement

*R*[*F* ^2^ > 2σ(*F* ^2^)] = 0.048
*wR*(*F* ^2^) = 0.135
*S* = 0.98
2834 reflections
180 parameters
H atoms treated by a mixture of independent and constrained refinement
Δρ_max_ = 0.24 e Å^−3^
Δρ_min_ = −0.16 e Å^−3^

### 

Data collection: *SMART* (Bruker, 1997 ▶); cell refinement: *SAINT* (Bruker, 1997 ▶); data reduction: *SAINT*; program(s) used to solve structure: *SHELXS97* (Sheldrick, 2008 ▶); program(s) used to refine structure: *SHELXL97* (Sheldrick, 2008 ▶); molecular graphics: *SHELXTL* (Sheldrick, 2008 ▶); software used to prepare material for publication: *SHELXTL*.

## Supplementary Material

Crystal structure: contains datablocks global, I. DOI: 10.1107/S1600536810022221/hb5490sup1.cif

Structure factors: contains datablocks I. DOI: 10.1107/S1600536810022221/hb5490Isup2.hkl

Additional supplementary materials:  crystallographic information; 3D view; checkCIF report

## Figures and Tables

**Table 1:** Hydrogen-bond geometry (Å, °)

*D*—H⋯*A*	*D*—H	H⋯*A*	*D*⋯*A*	*D*—H⋯*A*
N1—H1*A*⋯O1	0.86	2.33	2.692 (2)	106
N1—H1*A*⋯O3	0.86	2.07	2.920 (2)	169
O3—H3*B*⋯N3^i^	1.00 (4)	1.99 (4)	2.980 (2)	172 (3)
O3—H3*C*⋯O2^ii^	0.88 (3)	1.98 (3)	2.848 (2)	171 (3)

Symmetry codes: (i) 
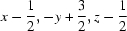
; (ii) 
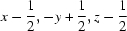
.

**Fig. 1.**
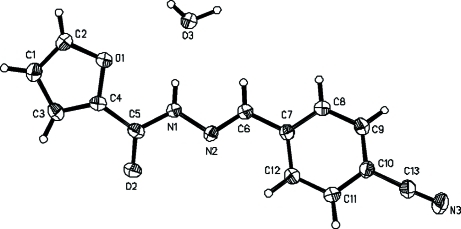
The structure of the title compound showing 30% probability displacement ellipsoids.

## supplementary crystallographic information

### Experimental

A mixture of 4-formylbenzonitrile (0.1 mol), and furan-2-carbohydrazide (0.1 mol) was stirred in refluxing ethanol (20 mL) for 2 h to afford the title compound (0.090 mol, yield 90%).  Colourless blocks of (I) were obtained by recrystallization from ethanol at room temperature.

### Refinement

H atoms were fixed geometrically and allowed to ride on their attached atoms, with C—H distances=0.97 Å, and with *U*_iso_=1.2–1.5*U*_eq_.

### Crystal data

**Table:**

C_13_H_9_N_3_O_2_·H_2_O	*F*(000) = 536
*M**_r_* = 257.25	*D*_x_ = 1.379 Mg m^−^^3^
Monoclinic, *P*2_1_/*n*	Mo *K*α radiation, λ = 0.71073 Å
Hall symbol: -P 2yn	Cell parameters from 1568 reflections
*a* = 7.0501 (14) Å	θ = 2.7–25.5°
*b* = 14.295 (3) Å	µ = 0.10 mm^−^^1^
*c* = 12.640 (3) Å	*T* = 293 K
β = 103.38 (3)°	Block, colorless
*V* = 1239.3 (4) Å^3^	0.22 × 0.20 × 0.18 mm
*Z* = 4	

### Data collection

**Table:**

Bruker SMART CCD diffractometer	1568 reflections with *I* > 2σ(*I*)
Radiation source: fine-focus sealed tube	*R*_int_ = 0.043
graphite	θ_max_ = 27.5°, θ_min_ = 3.3°
φ and ω scans	*h* = −8→9
11389 measured reflections	*k* = −18→18
2834 independent reflections	*l* = −16→16

### Refinement

**Table:**

Refinement on *F*^2^	Primary atom site location: structure-invariant direct methods
Least-squares matrix: full	Secondary atom site location: difference Fourier map
*R*[*F*^2^ > 2σ(*F*^2^)] = 0.048	Hydrogen site location: inferred from neighbouring sites
*wR*(*F*^2^) = 0.135	H atoms treated by a mixture of independent and constrained refinement
*S* = 0.98	*w* = 1/[σ^2^(*F*_o_^2^) + (0.0694*P*)^2^] where *P* = (*F*_o_^2^ + 2*F*_c_^2^)/3
2834 reflections	(Δ/σ)_max_ < 0.001
180 parameters	Δρ_max_ = 0.24 e Å^−^^3^
0 restraints	Δρ_min_ = −0.16 e Å^−^^3^

### Special details

**Table:**

Geometry. All e.s.d.'s (except the e.s.d. in the dihedral angle between two l.s. planes) are estimated using the full covariance matrix. The cell e.s.d.'s are taken into account individually in the estimation of e.s.d.'s in distances, angles and torsion angles; correlations between e.s.d.'s in cell parameters are only used when they are defined by crystal symmetry. An approximate (isotropic) treatment of cell e.s.d.'s is used for estimating e.s.d.'s involving l.s. planes.
Refinement. Refinement of *F*^2^ against ALL reflections. The weighted *R*-factor *wR* and goodness of fit *S* are based on *F*^2^, conventional *R*-factors *R* are based on *F*, with *F* set to zero for negative *F*^2^. The threshold expression of *F*^2^ > σ(*F*^2^) is used only for calculating *R*-factors(gt) *etc*. and is not relevant to the choice of reflections for refinement. *R*-factors based on *F*^2^ are statistically about twice as large as those based on *F*, and *R*- factors based on ALL data will be even larger.

### Fractional atomic coordinates and isotropic or equivalent isotropic displacement parameters (Å^2^)

**Table:**

	*x*	*y*	*z*	*U*_iso_*/*U*_eq_	
N2	0.2572 (2)	0.42473 (10)	0.51829 (12)	0.0431 (4)	
N1	0.2142 (2)	0.34390 (9)	0.45997 (12)	0.0440 (4)	
H1A	0.1704	0.3458	0.3906	0.053*	
O1	0.10154 (18)	0.19128 (9)	0.33670 (10)	0.0526 (4)	
C6	0.2239 (3)	0.50009 (12)	0.46228 (15)	0.0461 (5)	
H6A	0.1747	0.4964	0.3875	0.055*	
C4	0.1844 (3)	0.17816 (13)	0.44486 (15)	0.0442 (5)	
O3	0.0883 (2)	0.37589 (11)	0.22607 (12)	0.0654 (5)	
C13	0.3865 (3)	0.85907 (14)	0.65549 (17)	0.0548 (5)	
O2	0.3075 (2)	0.25365 (9)	0.61179 (11)	0.0595 (4)	
C12	0.3138 (3)	0.60216 (13)	0.62661 (16)	0.0525 (5)	
H12A	0.3218	0.5497	0.6709	0.063*	
C7	0.2620 (3)	0.59186 (12)	0.51417 (15)	0.0420 (4)	
C5	0.2414 (3)	0.26093 (12)	0.51272 (15)	0.0436 (4)	
C8	0.2453 (3)	0.67132 (13)	0.44979 (16)	0.0518 (5)	
H8A	0.2069	0.6655	0.3746	0.062*	
C11	0.3534 (3)	0.68908 (13)	0.67294 (16)	0.0551 (5)	
H11A	0.3887	0.6952	0.7482	0.066*	
C10	0.3405 (3)	0.76786 (12)	0.60708 (16)	0.0453 (5)	
C2	0.0589 (3)	0.10477 (13)	0.29261 (18)	0.0566 (6)	
H2B	0.0003	0.0931	0.2200	0.068*	
N3	0.4234 (3)	0.93164 (12)	0.69148 (17)	0.0721 (6)	
C1	0.1133 (3)	0.03943 (15)	0.36854 (18)	0.0654 (6)	
H1B	0.1006	−0.0249	0.3588	0.078*	
C9	0.2850 (3)	0.75903 (13)	0.49571 (16)	0.0521 (5)	
H9A	0.2741	0.8118	0.4516	0.062*	
C3	0.1943 (3)	0.08674 (13)	0.46686 (18)	0.0617 (6)	
H3A	0.2449	0.0593	0.5342	0.074*	
H3B	0.025 (5)	0.438 (3)	0.208 (3)	0.156 (14)*	
H3C	−0.005 (4)	0.337 (2)	0.197 (3)	0.120 (12)*	

### Atomic displacement parameters (Å^2^)

**Table:**

	*U*^11^	*U*^22^	*U*^33^	*U*^12^	*U*^13^	*U*^23^
N2	0.0504 (9)	0.0360 (8)	0.0404 (9)	0.0034 (7)	0.0056 (7)	−0.0056 (7)
N1	0.0572 (9)	0.0360 (8)	0.0346 (9)	0.0018 (7)	0.0023 (7)	−0.0016 (6)
O1	0.0708 (9)	0.0435 (7)	0.0389 (8)	−0.0040 (7)	0.0030 (6)	0.0018 (6)
C6	0.0560 (11)	0.0405 (10)	0.0384 (11)	0.0028 (9)	0.0037 (8)	−0.0023 (8)
C4	0.0466 (10)	0.0473 (11)	0.0366 (11)	0.0005 (9)	0.0052 (8)	0.0017 (8)
O3	0.0897 (11)	0.0468 (9)	0.0497 (9)	−0.0047 (9)	−0.0044 (8)	0.0021 (7)
C13	0.0600 (12)	0.0438 (11)	0.0576 (13)	0.0031 (10)	0.0073 (10)	−0.0013 (10)
O2	0.0829 (10)	0.0532 (8)	0.0369 (8)	0.0059 (7)	0.0026 (7)	0.0064 (6)
C12	0.0739 (14)	0.0408 (10)	0.0418 (11)	0.0062 (10)	0.0114 (10)	0.0024 (8)
C7	0.0435 (10)	0.0381 (10)	0.0428 (11)	0.0050 (8)	0.0072 (8)	−0.0002 (8)
C5	0.0451 (10)	0.0468 (11)	0.0377 (11)	0.0064 (9)	0.0074 (8)	0.0064 (8)
C8	0.0653 (12)	0.0467 (11)	0.0392 (12)	0.0028 (9)	0.0038 (9)	−0.0003 (8)
C11	0.0768 (14)	0.0483 (11)	0.0369 (11)	0.0042 (10)	0.0064 (10)	−0.0036 (9)
C10	0.0469 (10)	0.0383 (10)	0.0490 (12)	0.0020 (8)	0.0077 (8)	−0.0048 (8)
C2	0.0708 (14)	0.0460 (11)	0.0488 (12)	−0.0087 (10)	0.0052 (10)	−0.0062 (9)
N3	0.0868 (14)	0.0448 (11)	0.0786 (14)	−0.0037 (10)	0.0064 (11)	−0.0129 (9)
C1	0.0812 (15)	0.0401 (11)	0.0656 (15)	−0.0037 (11)	−0.0021 (12)	−0.0018 (10)
C9	0.0625 (12)	0.0396 (10)	0.0513 (13)	0.0008 (9)	0.0075 (10)	0.0067 (9)
C3	0.0757 (14)	0.0430 (11)	0.0575 (14)	0.0020 (10)	−0.0030 (11)	0.0125 (9)

### Geometric parameters (Å, °)

**Table:**

N2—C6	1.281 (2)	C12—C11	1.374 (3)
N2—N1	1.3664 (18)	C12—C7	1.391 (3)
N1—C5	1.352 (2)	C12—H12A	0.9300
N1—H1A	0.8600	C7—C8	1.386 (2)
O1—C2	1.361 (2)	C8—C9	1.383 (2)
O1—C4	1.370 (2)	C8—H8A	0.9300
C6—C7	1.464 (2)	C11—C10	1.391 (3)
C6—H6A	0.9300	C11—H11A	0.9300
C4—C3	1.335 (2)	C10—C9	1.377 (3)
C4—C5	1.462 (2)	C2—C1	1.331 (3)
O3—H3B	1.00 (4)	C2—H2B	0.9300
O3—H3C	0.87 (3)	C1—C3	1.414 (3)
C13—N3	1.139 (2)	C1—H1B	0.9300
C13—C10	1.445 (3)	C9—H9A	0.9300
O2—C5	1.235 (2)	C3—H3A	0.9300
			
C6—N2—N1	115.07 (15)	C9—C8—C7	121.00 (18)
C5—N1—N2	119.16 (15)	C9—C8—H8A	119.5
C5—N1—H1A	120.4	C7—C8—H8A	119.5
N2—N1—H1A	120.4	C12—C11—C10	119.89 (18)
C2—O1—C4	106.68 (15)	C12—C11—H11A	120.1
N2—C6—C7	121.01 (17)	C10—C11—H11A	120.1
N2—C6—H6A	119.5	C9—C10—C11	120.09 (17)
C7—C6—H6A	119.5	C9—C10—C13	119.90 (17)
C3—C4—O1	109.38 (16)	C11—C10—C13	120.01 (18)
C3—C4—C5	132.55 (18)	C1—C2—O1	110.06 (19)
O1—C4—C5	118.07 (15)	C1—C2—H2B	125.0
H3B—O3—H3C	102 (3)	O1—C2—H2B	125.0
N3—C13—C10	178.5 (2)	C2—C1—C3	106.77 (19)
C11—C12—C7	120.69 (17)	C2—C1—H1B	126.6
C11—C12—H12A	119.7	C3—C1—H1B	126.6
C7—C12—H12A	119.7	C10—C9—C8	119.63 (17)
C8—C7—C12	118.66 (16)	C10—C9—H9A	120.2
C8—C7—C6	119.31 (17)	C8—C9—H9A	120.2
C12—C7—C6	122.03 (16)	C4—C3—C1	107.11 (19)
O2—C5—N1	123.42 (16)	C4—C3—H3A	126.4
O2—C5—C4	120.96 (16)	C1—C3—H3A	126.4
N1—C5—C4	115.60 (16)		

### Hydrogen-bond geometry (Å, °)

**Table:**

*D*—H···*A*	*D*—H	H···*A*	*D*···*A*	*D*—H···*A*
N1—H1A···O1	0.86	2.33	2.692 (2)	106
N1—H1A···O3	0.86	2.07	2.920 (2)	169
O3—H3B···N3^i^	1.00 (4)	1.99 (4)	2.980 (2)	172 (3)
O3—H3C···O2^ii^	0.88 (3)	1.98 (3)	2.848 (2)	171 (3)

Symmetry codes: (i) *x*−1/2, −*y*+3/2, *z*−1/2; (ii) *x*−1/2, −*y*+1/2, *z*−1/2.
